# Are Non-Contact Thermometers an Option in Anaesthesia? A Narrative Review on Thermometry for Perioperative Medicine

**DOI:** 10.3390/healthcare10020219

**Published:** 2022-01-24

**Authors:** Andre van Zundert, Tonchanok Intaprasert, Floris Wiepking, Victoria Eley

**Affiliations:** Department of Anaesthesia and Perioperative Medicine, Royal Brisbane and Women’s Hospital, Brisbane and the University of Queensland, Brisbane 4072, Australia; t.intaprasert@uq.net.au (T.I.); floris@wiepking.org (F.W.); v.eley@uq.edu.au (V.E.)

**Keywords:** thermometry, thermometers, perioperative medicine, anaesthesia, infrared thermometer, contact thermometer, non-contact thermometer, body temperature, temperature, COVID-19

## Abstract

Measurement of core body temperature—clinical thermometry—provides critical information to anaesthetists during perioperative care. The value of this information is determined by the accuracy of the measurement device used. This accuracy must be maintained despite external influences such as the operating room temperature and the patient’s thermoregulatory defence. Presently, perioperative thermometers utilise invasive measurement sites. The public health challenge of the COVID-19 pandemic, however, has highlighted the use of non-invasive, non-contact infrared thermometers. The aim of this article is to review common existing thermometers used in perioperative care, their mechanisms of action, accuracy, and practicality in comparison to infrared non-contact thermometry used for population screening during a pandemic. Evidence currently shows that contact thermometry varies in accuracy and practicality depending on the site of measurements and the method of sterilisation or disposal between uses. Despite the benefits of being a non-invasive and non-contact device, infrared thermometry used for population temperature screening lacks the accuracy required in perioperative medicine. Inaccuracy may be a consequence of uncontrolled external temperatures, the patient’s actions prior to measurement, distance between the patient and the thermometer, and the different sites of measurement. A re-evaluation of non-contact thermometry is recommended, requiring new studies in more controlled environments.

## 1. Introduction

Monitoring of the five important vital signs—heart rate, blood pressure, respiratory rate, oxygen saturation, and temperature [1,2,3]—allow accurate diagnosis and treatment of pathological conditions. It is now common to find many instruments which monitor these vital signs available commercially for use at home [4]. The COVID-19 pandemic has seen significant expansion in the use of infrared thermometry, with thermometers used to detect individuals who are febrile, a common sign of the SARS-CoV-2 virus infection [5]. To meet this massive demand, a large number of non-contact infrared thermometers have entered the market [6]. If proven to be reliable and accurate, the use of these thermometers may be translated to the clinical setting, allowing non-invasive and non-contact monitoring of core body temperature.

Accurate thermometry is vital in the hospital setting to allow the effective diagnosis and treatment of medical conditions. During anaesthesia and postoperative recovery, anaesthetists strive to maintain normothermia in their patients, knowing that hypothermia is associated with increased wound infection, length of hospitalisation, intraoperative blood loss, post-operative discomfort, stress response, cardiac events, and morbidity [7,8,9]. Accurate and timely detection of hyperthermia is also essential to permit the diagnosis of life-threatening malignant hyperthermia [8,9]. Traditional non-contact thermometry devices in hospitals, however, have issues with accuracy and provide intermittent measurements [3]. Thus, current methods utilised in the perioperative setting are predominantly invasive contact devices inserted or measured directly at sites such as the bladder, nasopharynx, and oesophagus [3]. These devices permit continuous measurements.

The aim of this article is to review the characteristics and properties of thermometers used in hospital settings, clinics, and public health efforts such as the COVID-19 pandemic. As the norm shifts to social distancing and a preference for non-contact devices, we evaluate existing thermometers with the intent of identifying alternative methods for temperature monitoring in perioperative care. Particularly, if accurate non-contact thermometers can be identified, institutions would reap the cost-benefits of equipment requiring less disinfection, maintenance, and consumables.

## 2. Methods

**Figure 1:**
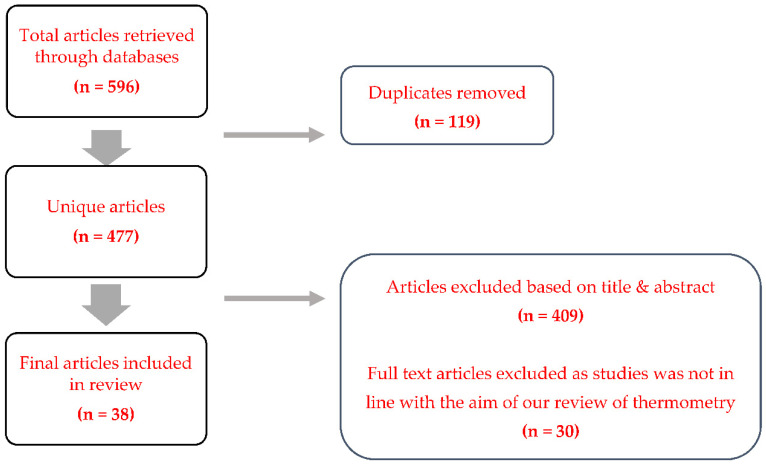
Results of the literature search and article screening.

We searched PubMed and Embase for articles published between 1 January 1980 and 1 August 2021 that were peer-reviewed in English. The following MeSH key words were used: thermo* AND perioperative AND body temperature. These yielded 201 articles from PubMed and 395 articles from Embase that were screened for titles, abstracts, and content (Figure 1). The ANZCA College library was also utilised for additional information. Articles discussing thermometry in perioperative medicine and non-contact thermometers in public health screenings were included in our final full text analysis. Studies where thermometry was mentioned in contexts other than perioperative medicine or public health in humans were excluded. The excluded studies were those that looked at consequences of hypothermia or methods to control body temperature rather than thermometry itself. Additional information on the working mechanics and properties of specific thermometers were retrieved directly from each manufacturer’s product information sheet. Altogether, we reviewed whether non-contact thermometry can influence our practices in anaesthesia based on current knowledge of perioperative temperature monitoring and studies of novel non-contact thermometers.

### 2.1. Body Temperature and Perioperative Monitoring

Hospitals utilise early warning scoring (EWS) systems to screen for derangements or deteriorations of patients [10]. The EWS comprises of multiple clinical observations and vital signs, one of which is body temperature. Human’s normal core body temperature ranges from 36.5 to 37.5 °C and averages around 37 °C [3]. These values are important as core temperature is considered the best indicator of thermal status [8]. Compared to temperatures measured from the skin and peripheries, core temperature around the head and thorax retains the most heat whilst thermoregulatory vasoconstriction maintains a gradient of cooler temperature towards the peripheries [11]. The hypothalamus then acts as the main thermoregulatory centre integrating thermal inputs and outputs to alter metabolic heat production and stabilise homeostatic thresholds within range [11]. The detection of hypothermia and hyperthermia is therefore an essential component of the EWS system.

During anaesthesia and surgery, patients are more at risk of developing changes to core body temperature [7,11]. The interthreshold range controlled by the hypothalamus is usually very small, tenths of a degree. Under general anaesthesia, the temperature threshold for vasoconstriction and shivering are lowered by 2–3 °C such that the interthreshold range is increased significantly and autonomic responses in general are impaired, reducing any thermoregulatory defence [11]. Both neuraxial and general anaesthesia cause vasodilatation, exacerbating the distribution of body heat from the core to the periphery and impairing the vasoconstrictive regulatory response. During anaesthesia, the patient may receive cold intravenous fluids, whilst also being exposed to cold ambient operating room temperatures with minimal clothing or coverings [9,12]. Combined with the absence of behavioural responses to hypothermia when anaesthetised, hypothermia during surgery is one of the most common temperature disturbances identified. In the post-anaesthesia care unit, hypothermic patients are more likely to experience thermal discomfort and shivering. These responses to hypothermia can interfere with pulse oximetry and non-invasive blood pressure measurement. Patients who experience intraoperative hypothermia are at risk of coagulopathy, wound infection, and myocardial ischemia [9]. Less commonly, hyperthermia can also occur during surgery. Hyperthermia can result from excessive warming, febrile infection, transfusion reactions, or malignant hyperthermia [3,8]. Thus, appropriate monitoring of temperature is vital to quickly and effectively detect and manage physiological changes during perioperative care.

Monitoring the patient’s temperature continuously during anaesthesia is ideal [13]; however, the suggested standard differs between guidelines. Recommendations from the National Institute for Clinical Excellence (NICE) suggests measuring the patient’s temperature one hour before induction and every 30 min intraoperatively, every 15 min in the Post Anaesthesia Care Unit (PACU), and every 4 h in the ward or every 30 min if active warming is required [11]. Similarly, the World Health Organization—World Federation of Societies of Anaesthesiologists (WHO-WFSA) recommends intermittent temperature monitoring and continuous monitoring only for certain cases [14]. The American Society of Anaesthesiologists (ASA) suggests monitoring when significant temperature change is anticipated or suspected, and the Association of Anaesthetists of Great Britain and Ireland (AAGBI) suggests monitoring when procedures last longer than 30 min in duration [14]. The Australian and New Zealand College of Anaesthetists (ANZCA), Canadian Anaesthesiologists’ Society (CAS), and the European Board of Anaesthesiology (EBA) recommend temperature monitoring to always be available with anaesthesia with a caveat from ANZCA stating that, in cases where warming devices are used, temperature monitoring is mandatory [14].

#### Monitoring Sites and Influences

As modern thermometers utilise different body sites and methods of measurement, levels of accuracy and sensitivity between devices vary. Core temperature in general is considered a more reliable indicator of thermal status than peripheral temperature. This is because core temperature is more tightly regulated compared to the periphery, which may vary considerably with environmental changes [8,11]. The pulmonary artery, brain, distal oesophagus, nasopharynx, and tympanic membrane are typically the sites accepted for accurate core temperature measures, while sites such as the mouth and axilla are considered “near-core” temperatures [7,8,15,16,17]. The bladder, rectum, and skin would be expected to give the least reliable core temperature [14]. This is because bladder and rectal temperatures show delayed changes to core temperature and the skin is most influenced by external exposures [7].

Many studies have compared different thermometer models against traditionally reliable core temperature sites and instruments [18,19,20,21,22,23]. Nonetheless, there is still no consensus nor universal guideline for what is the best site or modality of temperature monitoring and management intraoperatively [11]. From a practical perspective, certain measurement sites may be excluded due to the nature of the surgery being undertaken. Physicians are therefore left to their own preferences in selecting a device and site of measurement based on equipment availability, type of surgery, and accessibility [11].

Currently, thermometers used for perioperative medicine often require invasive contact measurement sites, such as in the nasopharynx or oesophagus. This either increases single-use equipment and environmental waste or increases the cost of sterilisation between uses. In addition, insertion of these devices is often limited to those with highly specialised skills. The potential benefits of the use of non-contact infrared thermometers in perioperative care warrants further consideration and may be preferable to the currently used specialised temperature measurement methods.

### 2.2. Overview of Thermometry

Scientists have dwelled over temperature for centuries and invented a multitude of measurement instruments for use in healthcare, household appliances, industrial settings, and much more. Thus, became the field of thermometry—thermal analysis involving the measurement of temperature over time [24]. Body temperature in particular has evolved from the subjective warmth of touch to the skin to quantitative measurements using thermometers [25]. Any instruments which measure heat transfer in the form of convection, conduction, or radiation are considered thermometers and the units of measurements may be Celsius, Fahrenheit, or Kelvin [26]. Measurement then results directly from temperature-sensitive transduction and electronic interpretation of the temperature change. In healthcare, the thermometers used quantify measurements after heat energy has been converted from transducers into a temperature scale. Thus, the sensing unit on a thermometer has characteristics that change with temperature variations. Traditionally, we can think of alcohol, mercury, and other fluids expanding in glass tube thermometers as examples [27]. More current sensing units, however, are made of different materials with thermal properties such as nickel and platinum.

#### 2.2.1. Common Sensing Unit

The mercury thermometer became unacceptable due to the risk of exposure to mercury, risk of injury from broken glass, the time taken to obtain a reading, and user variability when interpreting results [3,8,27]. Modern devices are more accurate and user-friendly, including the thermistor, thermocouple, resistance temperature detector, and infrared thermopile [8,15,28,29]. In anaesthesia, thermistors and thermocouples are the most common electronic sensing units used as both are inexpensive and sufficiently accurate [8].

**Thermistors** utilise semi-conductors which create variable resistance based on changing temperatures [8]. The materials can be subcategorised into those with negative or positive temperature coefficients [15]. Negative temperature coefficient materials such as thermistors made with oxides of iron, copper, and nickel show an inverse resistance to the changing temperature, whereas those of positive temperature coefficient will show a directly proportional resistance to temperature [15]. In anaesthesia, negative temperature coefficient thermistors, such as the YSI 400 series thermistor (Table 1), are most commonly used [30].

**Thermocouples** are composed of two different metal wires joined at one end to create a voltage difference that increases with temperature [8]. These thermometers are mathematically calibrated for the metal’s thermoelectric voltage corresponding to temperature differences [15]. A thermocouple’s metals, however, can corrode over time and affect sensor accuracy [15]. Thus, medical thermocouple probes may be best used as disposable devices to bypass the need for recalibration.

**Resistance temperature detector (RTD)** is often used in laboratories to calibrate for thermistors and thermocouples [28,31]. It is comprised of wires wrapped around a ceramic or glass core and temperature can be measured from the changes in resistance of its elements [15]. Platinum is most often used, while nickel and copper offer a lower cost but less stable alternative [31]. The platinum RTD is known for its accurate sensors in industrial applications and can cover a wide range of temperature spectrum from −200 to 800 °C with a fast response time. However, RTDs are costly and are not normally used as sensor units for individual body temperature thermometers [31].

**Thermopile infrared sensors** detect radiation at a distance and convert energy into temperature outputs [23,29]. Objects naturally emit infrared (IR) energy that increases with a rise in the object’s temperature [29] and IR sensors can detect this. These sensors detect body radiation within the electromagnetic spectrum for IR lying halfway between visible and microwave energy [3,32,33]. These waves are invisible to the human eye and are similar to radiation from the sun, fire, and radiator heat spectrum [33,34,35]. The ability to detect radiation at a distance makes these sensors perfect for use in healthcare situations where avoidance of body fluid contact and contamination is a priority. However, current utilisation has been mainly for public health screenings and infectious diseases, whereas we are interested in its accuracy and applicability for use in perioperative medicine. Therefore, we turn our attention to how these different sensing units are incorporated into different medical thermometers based on suitability.

#### 2.2.2. Common Clinical Thermometers

**Figure 2:**
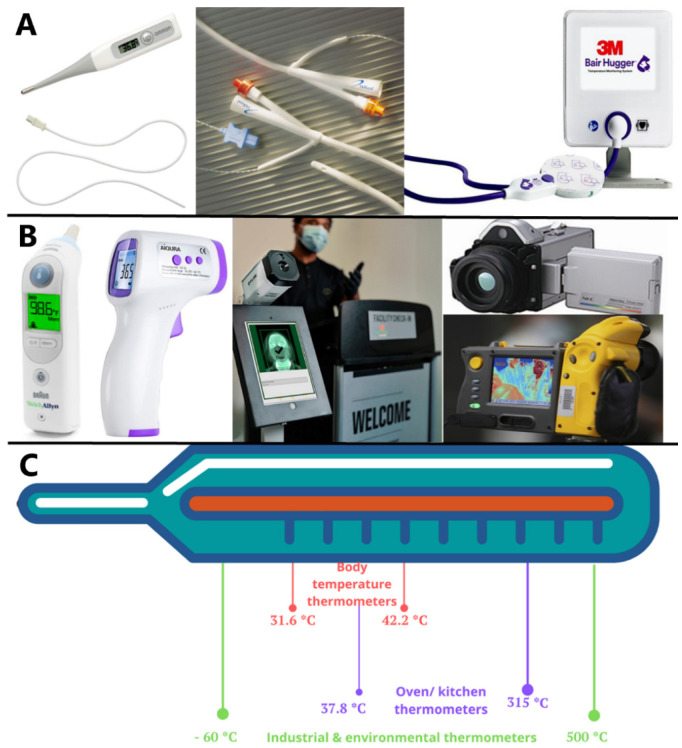
(**A**) Digital electrical thermometry: handheld thermometry and general-purpose catheter probe, zero-heat-flux thermometer; (**B**) various infrared thermometers (ear, forehead, one-person/mass screening); and (**C**) average temperature range measured by thermometers used for different purposes [40,41].

**Digital thermometers** are commonly used in clinical and household environments. When used at home, they are often handheld with a digital screen to convey measured temperature (Figure 2). In the hospital, these thermometers can be handheld or incorporated into bedside monitoring or portable systems. In perioperative monitoring, these thermometers utilise contact probes attached to an anaesthesia delivering device. The tip of these thermometers is made of thermistors or thermocouples [15]. They can be used to measure from multiple sites of the body and may include other parameters of measurement, such as pressure, brain tissue oxygen, and heart rate, for extended monitoring of patients [15]. Their probes can be placed within most sites considered accurate for core body temperature (i.e., oesophagus, brain, pulmonary artery) [7,15]. Therefore, with the hypothalamus considered the centre of core temperature regulation and the pulmonary artery the “gold standard” measurement site, digital thermometers are heavily relied upon in perioperative medicine for accurate thermometry [8].

**Zero-heat-flux thermometer** is used perioperatively and is a stick-on device which creates insulation between the thermometer insulator and the skin so there is theoretically no temperature gradient or flow of heat [3]. It achieves this by placing a layer of thermometer between skin and insulator, followed by another thermometer between the upper surface of the insulator and a heater [3]. The heater’s temperature helps eliminate any temperature gradient, and therefore heat flow, by maintaining both thermometer layers at the same temperature [3]. As core temperature starts to dissipate from the skin surface, it is trapped under insulation. After equilibrium, a column of tissue with core temperature will extend to the skin below the thermometer for measurement [3]. As this insulation is affected by lateral heat convection in blood flow, the ideal area of measurement is where the core is within a few centimetres of skin surface, such as the forehead and sternum [3]. A study comparing the zero-heat-flux thermometer to the distal oesophageal thermistor probe showed that, when measured at the sternum, its accuracy was adequate for clinical temperature monitoring [8].

**Electronic ear thermometers** are a common device in general practice and perioperative settings. They use bundles of optic fibre IR sensors to convert IR thermal energy to electric temperature signals using thermopiles or other similar sensors [8,28]. These thermometers are popular as they are easy to read, work fast, and have disposable sheaths that are easy to remove and replace between patients [28]. The sensor end is inserted into the ear adjacent to the tympanic membrane and within seconds the temperature is displayed on a digital screen [28]. As the tympanic membrane is considered an acceptable core temperature measurement site [11,14], the popularity of electronic ear thermometers has increased. In perioperative use, IR ear thermometers are often used postoperatively for monitoring of body temperature at intervals. A study by Bock et al. comparing its use in cardiac surgery to pulmonary artery thermometers also showed promising results for its use during surgery [36]. However, the accuracy of this method is still in question as most standard electronic ear thermometers available are unable to be placed physically close enough to the tympanic membrane [3,27].

**Other infrared thermometers (IRT)** utilise sensory and transduction principles converting IR radiation to temperature readings [15]. Similar to electronic ear thermometers, once IR radiation is detected, thermopiles convert the energy into temperature readings. The accuracy of these readings relative to the core temperature is influenced by the body site the temperature was taken, as heat is not emitted equally throughout the body. Two common sites targeted for measurements with IRT are the tympanic membrane and the forehead [11,29,35] (Figure 2).

However, recent studies have indicated that other measurement sites such as the inner canthus of the eye and over the temporal artery may be more reliable [15,37,38]. The use of IRT as a non-invasive alternative to traditional contact thermometer probes has been extensively studied. However, most current literature compares different types of IRT at different sites of the body with different contact thermometers measuring at differing sites—some accurate for core temperature while others are unreliable. Consequently, evidence for use of IRT instead of contact thermometers in perioperative medicine are inconclusive due to the variability of study methods in current literature.

**Thermography** is the visualisation of thermal radiation used to provide pictures of skin surfaces as a temperature map [15,29] (Figure 2). Like infrared thermometers, it is non-contact and safe [15]. Thermography allows the visualisation of temperature distribution on exposed skin surface as affected by blood flow and metabolic changes [15]. Thus, a mapping of inflammation, self-monitoring of diabetic foot, or evaluation of perfusion after procedures such as a free-flap surgery are possible [3,15]. It may also be used as an alternative to mammography for breast cancer screenings, although evidence of its sensitivity is still questionable [15].

During the COVID-19 pandemic and previous infectious disease outbreaks, mass screenings using infrared thermal imaging and thermography is common. Building on the technology used for infrared detectors in military applications, thermography improved from simple thermal detection to calibration of actual temperature ranges [39]. These improvements allow the technology for use in clinical medicine and public health. At major travel sites, such as airports, thermal scanners detecting raised temperatures range in sensitivity and specificity with a fairly low positive predictive value [15]. Thus, current infrared thermal imaging for the purpose of mass screenings simply offers a first line tool but cannot replace traditional thermometer or individually calibrated infrared and digital thermometers. For optimal patient outcome, the selection of the most appropriate thermometry device and measurement site needs to be individualised.

## 3. Standards for Thermometers

Currently, the accredited traceable calibration of temperature standard used for clinical thermometer is the International Temperature Scale of 1990 (ITS-90) [27,42]. This is to ensure that the thermometer is performing well and the temperature reading is traceable to the thermometer. Traceability refers to the fact that a reference standard thermometer calibrated in laboratories for working standard thermometers is used to calibrate thermometers used in clinical practice—and that this chain should be traceable [27]. These laboratories are accredited by a third party that checks technical and management aspects against international standard such as the International Organization for Standardization ISO/IEC 17025:2017 [27]. For medical electrical clinical thermometers, ISO 80601-2-56:2017 applies. For febrile human screening thermographs in a controlled environment, ISO 80601-2-59:2017 applies. The frequency at which thermometers need calibration depends on the stability of the thermometer type. A thermometer with well-known long-term stability will not require calibration as often as others with poor stability [27]. The range of acceptable errors for clinical thermometry should not exceed ±0.5 °C from true core temperature [3]. This range of inaccuracy is the maximum acceptable for the combined thermometer and site error in core body temperature measurement [3,8]. However, most inaccuracies in clinical thermometry stem from the site of measurement rather than the device itself [3].

Recent use of thermal imaging to screen for fevers during the COVID-19 pandemic emphasised the importance of thermometer range and accuracy even further. The ISO states that the standard for use of thermal imaging to screen for true indication or absence of fever is with a close-up image of the face where a minimum of 9 pixels can be located in both inner canthi [39]. However, current use in some airports, shopping centres, and other public gatherings tends to evaluate large groups of moving people from a distance. This method, also used during the SARS outbreak in 2003, was found to be ineffective because individuals can be febrile without a generalised increase in facial temperature [39]. Thus, the use of thermography for accurate body temperature measurement is still uncertain and its application in perioperative medicine remains unknown.

## 4. Perioperative Thermometers vs. COVID-19 Fever Screening IRT

The main similarity and differences of IRT and traditional contact thermometers can be seen in Table 1. IRT and thermography offers the advantages of being non-contact with an easy set up for continuous monitoring. It is overall less invasive and does not require a skilled physician for insertion into invasive body sites. However, the literature to date shows conflicting evidence for IRT and thermography in febrile screenings. Studies evaluated their accuracy for use in airports and public spaces [43], which are not equivalent to the relatively controlled operating theatre environment. Thus, external factors influencing body temperature readings (i.e., ambient temperatures, recent exercise, diurnal patterns) were not controlled for. Infrared thermometers for use in clinical settings should therefore not yet be excluded.

Recent meta-analyses of non-core temperature site monitoring conclude that only oral and rectal digital thermometers are acceptable for alternate use in screening, monitoring, diagnosing, and treating patients [44,45]. However, only tympanic and temporal artery IRT were included in the study, excluding other IR thermometer types. Furthermore, the paper identified tympanic, temporal artery, and axillary thermometers as inaccurate for monitoring core body temperature [44]. These results are in conflict with current understanding of accurate measurement sites [8]. It is likely that differences in accuracy between different models of thermometer may play a role in measurement accuracy. As oral and rectal sites are sometimes unavailable in perioperative settings, and other measurement sites more invasive, further evaluation continues to determine if IRT and thermography is appropriate for translation into perioperative medicine.

A recent study by Chan et al. evaluated the use of infrared thermography in the ICU [46]. This research is promising as it looks at the use of infrared cameras outside of public health screenings. The results from this study showed that the thermal imaging camera used did not measure the patient’s temperature at the clinical standards required in the ICU [46]. Similar clinical studies with other non-contact thermometer models and expansion of testing to the operating theatre environment may help identify features to improve infrared thermography’s accuracy or definitively advise against its use in hospital medicine. Most current literature compares different types of non-contact thermometers with different models of contact thermometers at differing sites of the body—some accurate for core temperature while others are unreliable. Evidence for use of IRT as an alternative to contact thermometers in anaesthesia is inconclusive due to the variability of study methods in current literature.

Future research should include analysis of the accuracy of these techniques under different conditions, which could be standardised in the operating theatre. Important elements to be considered include sites of measurement, optimal distance of measurement, controlled external temperatures and patient’s movements, the ability to capture hypothermia as well as hyperthermia, and closeness to core body temperature when measured within perioperative settings.

## 5. Limitations

Due to a lack of available literature on IRT and thermography for body temperature monitoring outside of public health use, a conclusion cannot be made on whether these technologies can be reliably used in perioperative medicine. However, due to its convenience and ability for continuous non-contact monitoring, it is worthwhile for clinicians and engineers to conduct further studies. These studies should standardise the thermometer model, brand, site of measurement, and environment for replicability and comparability. As thermometry studies start to control for more similar variables, literature can increase in robustness as more information becomes available on each device model with a better comparison of current technology.

Thermometry as a discipline encompasses a wide range of industries. Anything requiring temperature to be measured or controlled utilises some form of thermometer device with different sensory probes and mechanisms. Thus, it was not feasible to include every model, sensory probe, or device from all industries in this review, which focused on thermometers commonly used in the perioperative setting and IRT for febrile screenings. It may be useful that further studies investigate the evolution and history of thermometry to determine the level of technological maturity and general acceptance of each device in the medical market.

## 6. Conclusions

Clinical thermometers for use in perioperative medicine should reflect accurate core body temperature, be able to reflect temperature changes without delay, and allow physicians to attend to any derangements in a timely manner. Current perioperative thermometers utilise thermometer probes requiring contact with patients. Some require invasive contact such as insertion into the nasopharynx or pulmonary artery. With the onset of COVID-19 and the increased usage of IRT and thermography for temperature screenings, their mechanisms and properties were investigated for suitability in perioperative medicine.

IRT and thermography are ideal for continuous non-contact uses. They require less sterilisation and cleaning and do not require invasive sites to monitor patient’s temperature. However, current studies available have only looked at its suitability for use in febrile screenings or as a diagnostic tool. There are currently limited studies looking at accuracy and feasibility of using these thermometers in perioperative temperature monitoring. Therefore, in a controlled operating theatre environment, further studies analysing the accuracy of IRT for core temperature measurement and its ability to distinguish temperature fluctuations may prove beneficial.

## Figures and Tables

**Table 1:** Properties and mechanisms of common contact thermometers, infrared thermometers, and thermographs.

Sensor Unit and Models	Working Mechanics	Calibration Frequency/Traceability	Accuracy *	Core/Peripheral Temperature	Contact (Y/N)	Invasive (Y/N/B)	Robustness	Consumables Used
**Thermistors**								
YSI 400 series Foley Catheter Temperature Sensor, DeRoyal©, DeBusk Lane Powell, TN, 37849, USA	Thermistor probe attached to catheter of multiple sizes	Single use, no recalibration	++++	Core	Y	B	Sterile single use	One use thermometer probe
YSI 400 and 700 series, Xylem Inc., Tokyo, Japan	Thermistor probe with multiple tip sizes, materials, and shapes for different sites	Traceable to US National Institute of Standards and Technology (NIST)	++++	Core	Y	B	Reusable—ethylene oxide gas sterilisation	Sterilisation materials
**Thermocouples**								
Thermocouple probes, Harvard Apparatus, MA, Hollistion, USA	Proprietary copper thermocouple wires with multiple tip sizes and shapes for different sites	No recalibration required	+++++	Core	Y	B	Reusable—gas/cidex sterilisation	Sterilisation materials
Level 1^®^ Temperature Monitoring Probes, SAN CLEMENTE ICU Medical, Inc. 951 Calle Amanecer San Clemente, 92673, CA, USA	Lead wire thermocouple probe with multiple tip sizes and shapes for different sites	Start-up standardise calibration to one monitoring system required	++	Core	Y	B	Reusable and pliable probes for sterilisation between use	Sterilisation materials
**Infrared**								
Braun ThermoScan^®^ PRO 6000, Welch Allyn, Southborough, MA, Hollistion, USA	Infrared proprietary sensory probe	Annual calibration check suggested	+++	Core	N	N	70% isopropyl or ethyl alcohol to clean probe lens window—needs to be maintained for accurate readings	Single-use disposable probe cover
Omron^®^ TH839S, HsinChu, Taiwan	Infrared thermopile detectors	No stated calibration frequency/traceability found	+++	Core	N	N	Delicate probes require care when cleaning	Single-use disposable probe cover
**Thermograph**								
FLIR Elara™ FR-345-EST, FLIR Systems, Inc., Wilsonville, 97070, CA, USA	Infrared thermal imaging microbolometer	Initial calibration on set up with recalibration if set up is disturbed by use or cleaning	+	Peripheral	N	N	Operates best in specific humidity, temperature, and distance to enhance accuracy	No consumables or sterilisation
InfReC R550series, Nippon Avionics Co., Ltd., Tokyo, Japan	Infrared thermal imaging or isotherm imaging	No stated calibration frequency/traceability found	−	Peripheral	N	N	Quite robust, only need upkeep of electronic accessory components	No consumables or sterilisation

* Accuracy range of measurement errors ±0.1 (+++++), ±0.1 to 0.2 (++++), ±0.2 (+++), ±0.3 (++), ±0.5 (+), 1 (−); Y = yes; N = no; B = both.

## Data Availability

Study did not report data.
